# Supplementary material for: Particulate Matter Emission Factors for Dairy Facilities and Cattle Feedlots during Summertime in Texas
Source: Int J Environ Res Public Health. 2022 Oct 28;19(21):14090. doi: 10.3390/ijerph192114090 (PMC9657701; doi:10.3390/ijerph192114090)
Supplement: Supplementary file 1 [file ijerph-19-14090-s001.zip › ijerph-1918008-supplementary.pdf]

Supplementary Document

Tables

Table S1. Surface parameters used for AERMOD

Category	Sector	Albedo	Bowen ratio	Surface Roughness
Cultivated Land	1	0.14	0.45	0.0725
Grass Land	2	0.14	0.45	0.04025

Table S2. Pearson Correlation for collocation of samplers using the PM2.5 emission measures from feedlot C.

	PM2.5		
	BAM	AEROCET	Drone AEROCET
BAM	1		
AEROCET	0.79	1	
Drone AEROCET	-0.02	0.31	1

Table S3. Benchmarking of calculated PM EFs against published EFs for US free-stall dairies.

	Annual Emission Factors (kg/1000hd/d)	
	PM2.5	PM10
Present Study	0.34	5.59
USEPA (1985)[a]	x	21
Parnell et al. (1994)	x	6.8
USDA, 2000 [b]	x	2.9 (dry lot)
Goodrich et al (2006)	x	37

[a] using 25% PM10/TSP ratio

[b] using 15% PM2.5/PM10 ratio

Table S4. Benchmarking of calculated PM EFs against published EFs for US beef cattle feedlots.

	Emission Factors (kg/1000hd/d)	
	PM2.5	PM10

Present Study	6.47	21.76
USEPA (1985)[a]	12	82
USDA, 2000 [b] (model farm dry feedlot)	x	15.8
Wanjura et al (2004)	x	19
Auvermann et al (2010)	x	71
Bonifacio et al (2015)	11	21

[a]estimated using scaling factors
[b] using 15% PM2.5/PM10 ratio

Figures

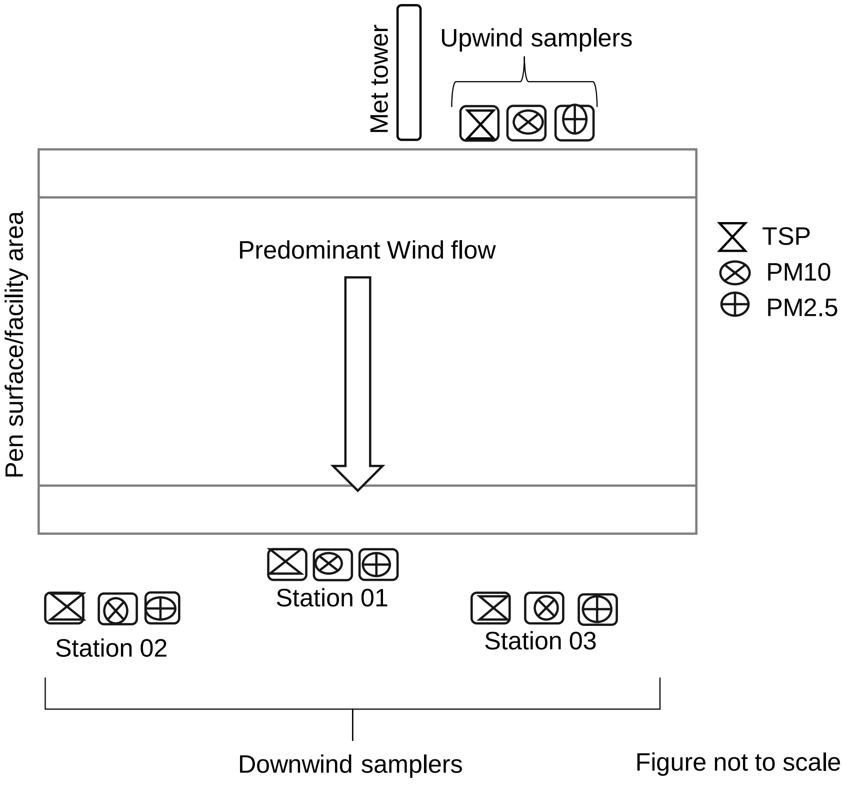

Figure S1. Sampling schematic set up for emission collection in the feedlot and the dairy.

Soil Particle Size Distribution (PSD)

Soil sample was collected from the dairy and feedlot and analyzed for particle size distribution. PSD of top soil can help interpret the emitted dust sizes from the environment. The dairy facility textural class was sandy loam soil with about 57% sand composition while the feedlot textural class was loamy sand with about 81% sand composition. The particle size distribution showed normal distribution for both the sampling locations (Fig. S2). The Scanning Electron Microscope (SEM) image (Fig. S3) shows the emitted particle's aerodynamic shape using the TESCAN VEGA3 SEM of TAMU Microscopy and Imaging Centre (MIC). The PM2.5 image was achieved with 8.0 kx SEM magnification and the PM10 image was achieved with 8.19 kx SEM magnification.

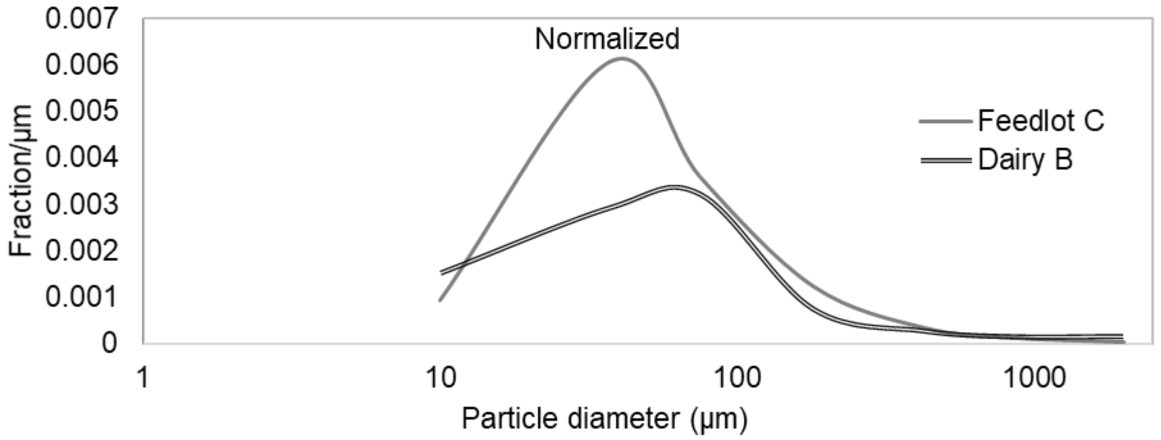

Figure S2. Normalized Particle Size Distribution (PSD) of soil samples from the Dairy and the Feedlot.

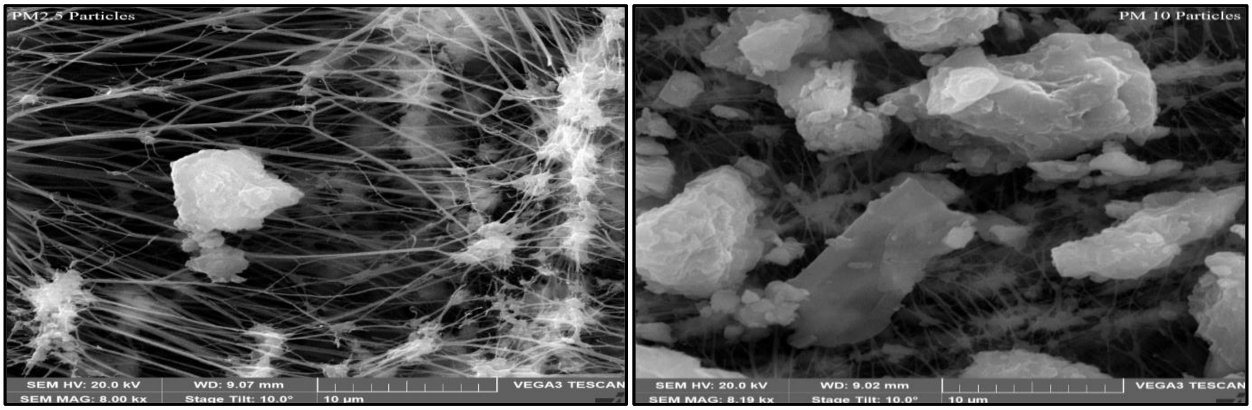

Figure S3. SEM image of aerodynamic shape for PM2.5 (left) and PM10 (right) of the collected samples.

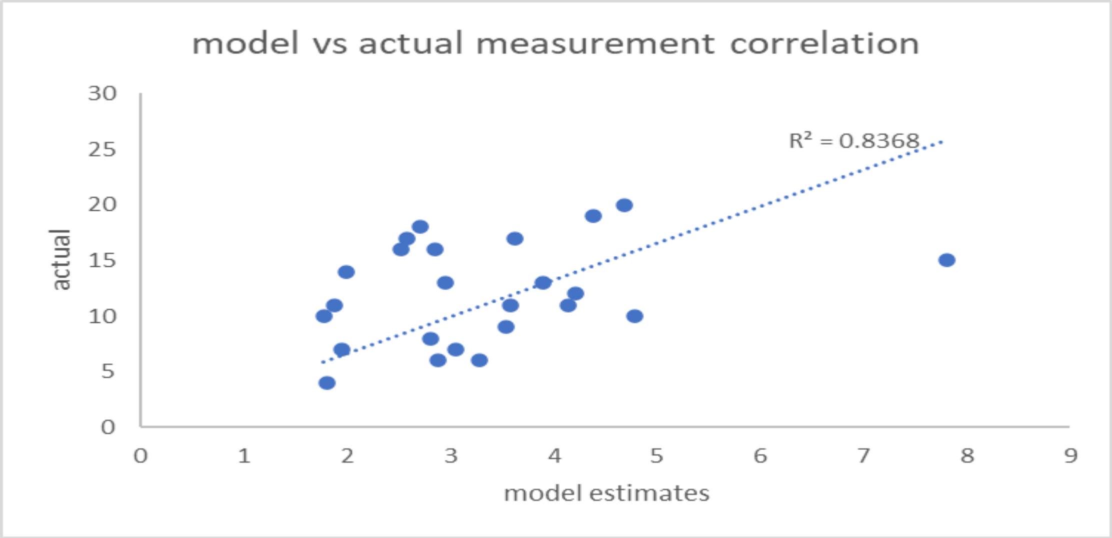

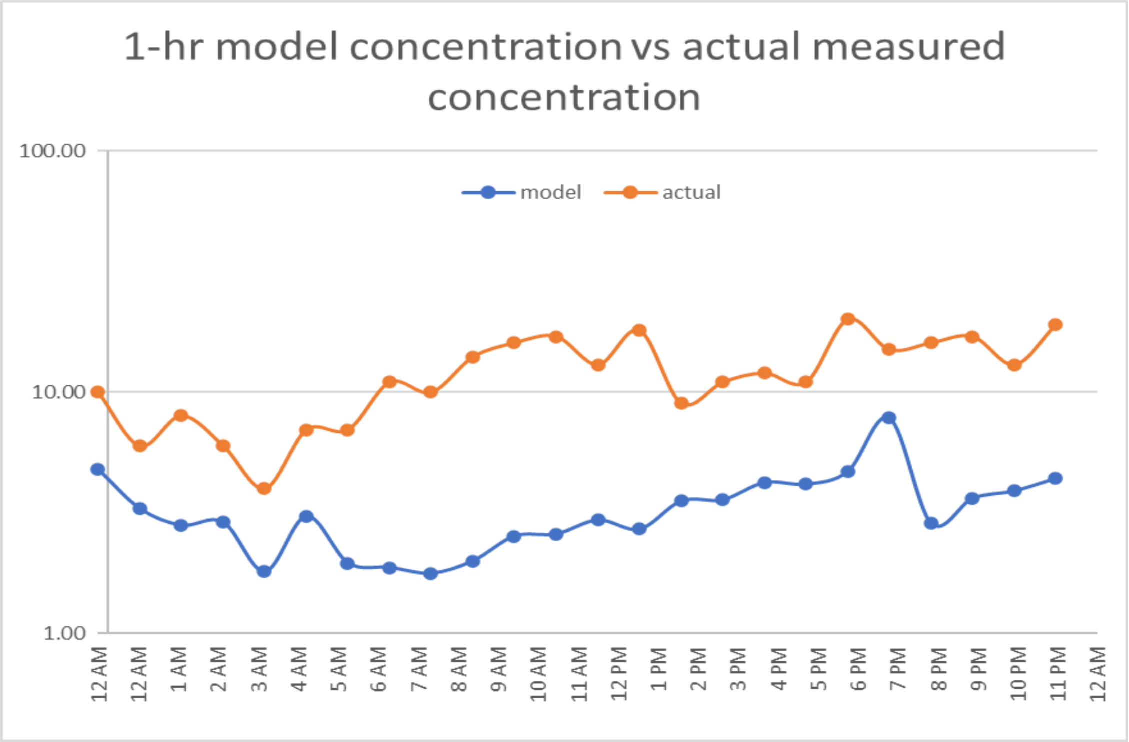

Figure S4. Correlation of the actual and AERMOD modelled 1-hr PM2.5 data concentration (μg m^{-3}) on a random day in the dairy.

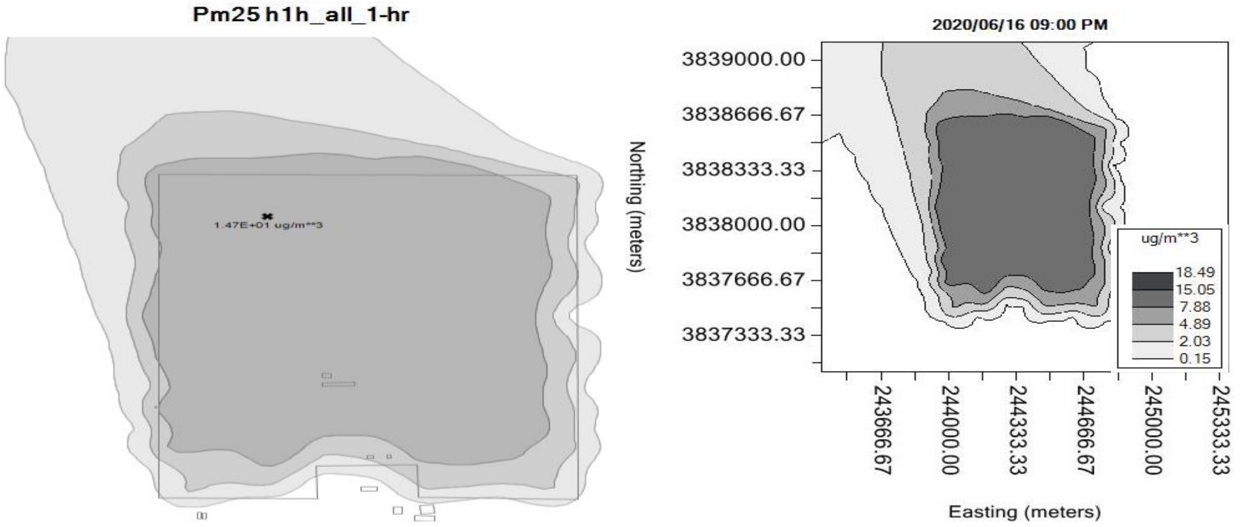

Figure S5. PM2.5 emission dispersion at the feedlot on June 16 at 9 pm and cross section view (right side)

More than 60% of the time, the PM emission dispersion in the feedlot was to the north, specifically northeast or northwest. Fig. S4 shows the PM2.5 dispersion from the feedlot on June 16 at 9 pm directing to the northwest. The soil of north side agricultural lands may exhibit repercussions from this PM dispersion containing high manure nutrients. All the downwind stations placed in the feedlot were in the second contour zone (15.1 μg m^{-3}) showing a high emission factor on June 16 at 9 pm. Higher night emissions may have been influenced by the animal activity favored by pleasant weather conditions.

References:

1. U.S. Environmental Protection Agency (USEPA). 1985. Compilation of Air Pollutant Emission Factors, AP–42. Stationary and Area Sources, vol. I, USEPA, Research Triangle Park, NC, USA (1985)
2. Parnell, S.E. (1994). Dispersion Modeling for Prediction of Emission Factors for Cattle Feedyards. Master’s Thesis, Texas A&M University. Available electronically from. https://hdl.handle.net/1969.1/ETD-TAMU-1994-THESIS-P256.
3. U.S. Department of Agriculture (USDA). 2000. Confined Livestock Air Quality Subcommittee, J. M. Sweeten, Chair. U.S. Department of Agriculture (USDA), Agricultural Air Quality Task Force (AAQTF) Meeting, Washington, DC. Air Quality Research & Technology Transfer Programs for Concentrated Animal Feeding Operations.
4. Goodrich, L.B. 2006. A PM10 emission factor for free stall dairies. Master's thesis, Texas A&M University. Texas A&M University. [Available electronically from https://hdl.handle.net/1969.1/3858
5. Bonifacio, H. F., Maghirang, R.G., Trabue, SL., McConnell, L.L., Prueger, J.H., Bonifacio, E.R. (2015). TSP, PM10, and PM2.5 emissions from a beef cattle feedlot

using the flux-gradient technique. Atmos. Environ., 101, 49-57. https://doi.org/10.1016/j.atmosenv.2014.11.017.

6. Auvermann, B.W, Bush, K.J., Marek, G.W., Heflin, K., Wilhite, W.B., Sakirkin, S.L.P. (2010). Time-varying PM10 emissions from open-lot dairies and cattle feedyards. Presented at the International Symposium on Air Quality and Manure Management for Agriculture, Dallas, Texas, September 13–16, 2010.
